# P-210. ASHAs as Agents of Change: Tackling the Burden of UTIs and Fostering Antibiotic Stewardship in Underserved Communities of India

**DOI:** 10.1093/ofid/ofaf695.432

**Published:** 2026-01-11

**Authors:** Sudarshan Hanumappa, Sanjana Ravi, Sumana Mahadevaiah, Sunitha Chandrasekhar Srinivas, Natalie Axelrod, Siri C Peddineni, Vaishnavi RV, E S Chinchana, G S Veerabhadraswamy, Gautam Kalyatanda, Chilsia Shafi, Venkat Chekuri

**Affiliations:** Karuna Trust - Bangalore India, Bangalore, Karnataka, India; University of Florida College of Medicine, Gainesville, FL; SS Health University - Mysuru India, Mysore, Karnataka, India; Karuna Trust, Bangalore, Karnataka, India; University of Florida College of Medicine, Gainesville, FL; University of Florida College of Medicine, Gainesville, FL; Karuna Trust, Bangalore, Karnataka, India; JSS Health University - Mysuru India, Mysore, Karnataka, India; JSS Health University - Mysuru India, Mysore, Karnataka, India; University of Florida, Gainesville, Florida; University of Florida College of Medicine, Gainesville, FL; Karuna Trust, Bangalore, Karnataka, India

## Abstract

**Background:**

Women living in slums in India often experience stigma when seeking treatment for urinary tract infections (UTIs), especially when care is provided by male healthcare workers at public primary health care (PHC) centers. To address this issue and other barriers, community health workers known as Accredited Social Health Activists (ASHAs) were leveraged to assist with outreach and improve access to UTI care.

**Methods:**

Educational sessions were held at eight Anganwadi centres (public child and mother-care centres) to educate women about urinary tract infections (UTIs), including their causes, symptoms, and the importance of treatment adherence.

Additionally, training sessions for Accredited Social Health Activists (ASHAs) focused on antimicrobial stewardship. ASHAs identified women with UTI symptoms, collected urine samples, and sent them to an accredited laboratory for analysis. If bacterial growth was confirmed, ASHAs helped these women access appropriate treatment by accompanying them to local primary health centres (PHCs).

**Results:**

Through active surveillance conducted by ASHAs, 54 women with urinary symptoms were identified. Among these, 31 samples were excluded from the study because they did not meet the inclusion criteria, specifically due to the absence of pyuria. Of the remaining 23 samples, 14 exhibited sterile pyuria, while 9 (40%) grew bacteria, which were subsequently treated with appropriate antimicrobials.

**Conclusion:**

This initiative underscores the essential role of last-mile health providers in addressing women’s health by enabling the timely identification and treatment of urinary tract infections (UTIs) through active surveillance. The project empowers Accredited Social Health Activists (ASHAs) to improve access to care for women who avoid seeking treatment due to stigma, while fostering trust and encouraging symptom reporting.

**Disclosures:**

All Authors: No reported disclosures

